# Identification of a Heat-Inducible Element of Cysteine Desulfurase Gene Promoter in *Lentinula edodes*

**DOI:** 10.3390/molecules24122223

**Published:** 2019-06-14

**Authors:** Zhicheng Huang, Xiaoyu Lei, Xi Feng, Shuangshuang Gao, Gangzheng Wang, Yinbing Bian, Wen Huang, Ying Liu

**Affiliations:** 1 College of Food Science and Technology, Huazhong Agricultural University, Wuhan 430070, China; HuangZhiCheng1210@163.com (Z.H.); xiaoyulei1988@126.com (X.L.); 13720161459@163.com (S.G.); huangwen@mail.hzau.edu.cn (W.H.); 2 Department of Nutrition, Food Science and Packaging, California State University, San Jose, CA 95192, USA; xi.feng@sjsu.edu; 3 Institute of Applied Mycology, Plant Science and Technology College, Huazhong Agricultural University, Wuhan 430070, China; wgzhau@163.com (G.W.); bianyinbing@mail.hzau.edu.cn (Y.B.)

**Keywords:** *Lentinula edodes*, gene promoter, volatile organosulfur compounds, heat-inducible element

## Abstract

Volatile organosulfur compounds are the main components that contribute to the unique aroma of dried *Lentinula edodes*. They are mainly generated during the hot-air drying process, and cysteine desulfurase is the key enzyme in this process. Temperature may be an essential factor of volatile organosulfur compound production by influencing the expression of the cysteine desulfurase gene. In this study, the promoter sequence of the cysteine desulfurase gene (*pCS*) was cloned and analyzed using bioinformatics tools. A series of 5′deletion fragments and site-directed mutations of *pCS* were constructed to identify the element that responds to heat stress. Six heat shock transcription factor (HSTF) binding sites were predicted by SCPD (The Promoter Database of *Saccharomyces cerevisiae*) and three of the binding sites were predicted by Yeastract (Yeast Search for Transcriptional Regulators and Consensus Tracking) in *pCS*. The results indicated that *pCS* was able to drive the expression of the *EGFP* (Enhanced Green Fluorescent Protein) gene in *L. edodes*. Moreover, the fluorescence intensity increased after heat stress. The changes in fluorescence intensity of different 5′deletion fragments showed that the heat response region was located between −500 bp and −400 bp in *pCS*. The site-directed mutation analysis further showed that the heat-inducible element was between −490 bp and −500 bp (TTTCTAGAAT) in *pCS*. Our results provide molecular insight for studying the formation of volatile organosulfur compounds in dried *L. edodes*.

## 1. Introduction

*Lentinula edodes* (shiitake mushroom), the second most cultivated edible mushroom in the world, has been used as food and traditional medicine for 2000 years [1]. Fresh mushrooms usually rapidly go through open-caps, browning or shrinking after harvesting, which cause the loss of commodity value and edible quality. Fresh mushrooms are often dried to preserve their quality and nutritional value. Moreover, the smell of dried *L. edodes* is one of the most important indexes to assess its quality, which is entirely different from fresh *L. edodes*. Therefore, dried *L. edodes* is especially prized because of its characteristic flavor [2]. Sulfur compounds including disulfide, trisulfide, 1,2,4-trithiolane, 1,2,4,6-tetrathione, and 1,2,3,5,6-pentathiepane (lenthionine) contribute the major flavors of dried *L. edodes* [3], while carbon-8 compounds are the major volatile compounds in fresh *L. edodes* [4]. Lenthionine, a cyclic sulfur compound, is the characteristic flavor component in dried *L. edodes* due to its low threshold value [5,6].

However, different drying methods significantly affect the flavor of dried *L. edodes*. Previous studies in our lab also showed that the volatile sulfide is significantly different during three drying methods including natural drying, hot-air drying, and freeze drying. Convective drying at 80 °C for 120 min has the highest contents of total volatiles and cyclic sulfur compounds [4,7]. Among all of the drying methods, hot-air drying is the most economical and suitable to produce dried *L. edodes* at the industrial scale. However, the mechanisms of drying temperature on the formation of volatile organosulfur compounds of *L. edodes* is still unknown.

Lentinic acid (γ-L-glutamyl-cysteine sulfoxide) is the precursor of lenthionine, and γ-glutamyl transpeptidase (GGT) and cysteine sulfoxide lyase (C-S lyase) are the two key enzymes in this reaction [8,9]. The content of lentinic acid increased significantly after a drying process at 40 °C for 4 h [10]. Recently, the C-S lyase in *L. edodes* was reported to be a novel cysteine desulfurase and not a type of cysteine sulfoxide lyase [11]. An exogenous expression system of the cysteine desulfurase gene (*Csl*) was constructed, and it obtained an active recombinant enzyme, which was found to catalyze lentinic acid to generate volatile organosulfur compounds [12]. However, there are few studies on the molecular function of cysteine desulfurase on the formation of volatile organosulfur compounds in *L. edodes*. In addition, the temperature may influence the formation of volatile organosulfur compounds in *L. edodes* by regulating the transcription level of the cysteine desulfurase gene during the drying process [13].

The heat shock element (HSE) is a specific sequence in the promoter region that binds with the heat shock transcription factor (HSTF/Hsf1) and promotes the transcription level of heat-inducible genes during the thermal resistance process. The HSE is a functional domain present in many genes such as *APX1* [14], *HSP* [15], *PGK* [16], and heat shock operon *danK* [17]. Thus, HSE or HSE-like elements may be the most important factors for the gene *Csl* to respond to heat stress during the hot-air drying process. The objectives of this study were to analyze the function of the promoter of *Csl* gene (*pCS*) in *L. edodes*.

## 2. Results

### 2.1. Bioinformatic Analysis of Putative Cis-Elements in pCS

The bioinformatics analysis showed that some putative elements were predicted in *pCS*. As shown in Table 1, 44 of elements in *pCS* were predicted by SCPD. An RNA polymerase II binding site TATA-Box (TATATA) was found to be located 57 bp upstream of the ATG sequence on both strands. More importantly, seven elements with homology to heat shock transcription factor (HSTF) binding sites (TTCAACGAA) were found which meant the gene *Csl* may be able to respond to heat stress. The *pCS* also contained one Cu^2+^ response element (GAGCAAA, −664), one element involved in DNA repair (CTTCCT, −1282), and one RNA polymerase I biding site (CCACCCG, −19). As shown in Table 2, 77 of the elements in *pCS* were predicted by Yeastract, and 43 of the elements were on the forward strand, whereas the others were on the opposite strand. Three of the heat shock factor (Hsf1) binding sites were found on both strands. Moreover, the positions of Hsf1 were consistent with three of the HSTF binding sites. A Cu^2+^ response element (TTTGCKCR, −636) also existed, while its position and the signal consensus in *pCS* were different compared with the results of the SCPD. Nine of the Gcr1p binding sites were predicted in *pCS*, whereas the Gcr1p transcriptional factor was involved in the regulation of glycolytic genes [18]. There were 27 of the predicted transcriptional binding sites, including nine of the Mpt3p, one of the Skn7p, 15 of the Stb5p, and two of the Yap1p which participated in oxidative stress. Tec1p was reported to be a transcription factor involved in the expression of hypha−specific genes in *Candida albicans* [19]. Two of Tec1p binding sites were predicted in *pCS*. Totally, three of the heat shock transcription factors binding sites were predicted by both Yeastract and SCPD, and the binding sites were located between −801 bp and −811 bp, −522 bp and −512 bp, and −490 bp and −500 bp.

### 2.2. EGFP Expression in Transformants Driven by Full-Length pCS

**Figure 1:**
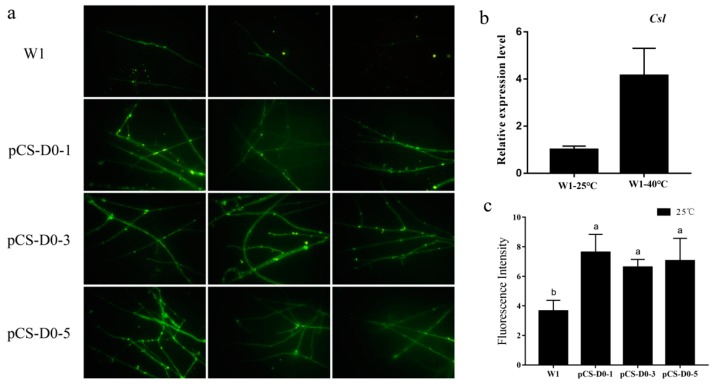
(**a**) Mycelia of pCS-D0 transformants observed with an OLYMPUS BX51 fluorescence microscope compared to wild-type W1; images were taken with a 40× field of view. (**b**) Relative expression level of *Csl* before and after heat stress in wild-type W1. (**c**) The fluorescence intensity of pCS-D0 transformants; *n* = 3, *p* < 0.01.

After being grown on MYG(Malt extract glucose) medium for 3 days, 13 of the transformants which expressed *EGFP* under *Csl* promoter were identified via PCR reaction with primers GPD-F/R. Three of the identified transformants named pCS-D0-1, pCS-D0-3, and pCS-D0-5 were selected for analysis using an OLYMPUS BX51 fluorescence microscope. The fluorescence intensity of each transformant was calculated and given by Image-Pro Express. As shown in Figure 1a, the wild-type strain W1 mycelia showed a weak fluorescence under 25 °C. However, the fluorescence intensity of the three transformants was two-fold higher than that of W1. Our results indicated that the promoter *pCS* were able to drive the expression of *EGFP* in *L. edodes*.

**Figure 2:**
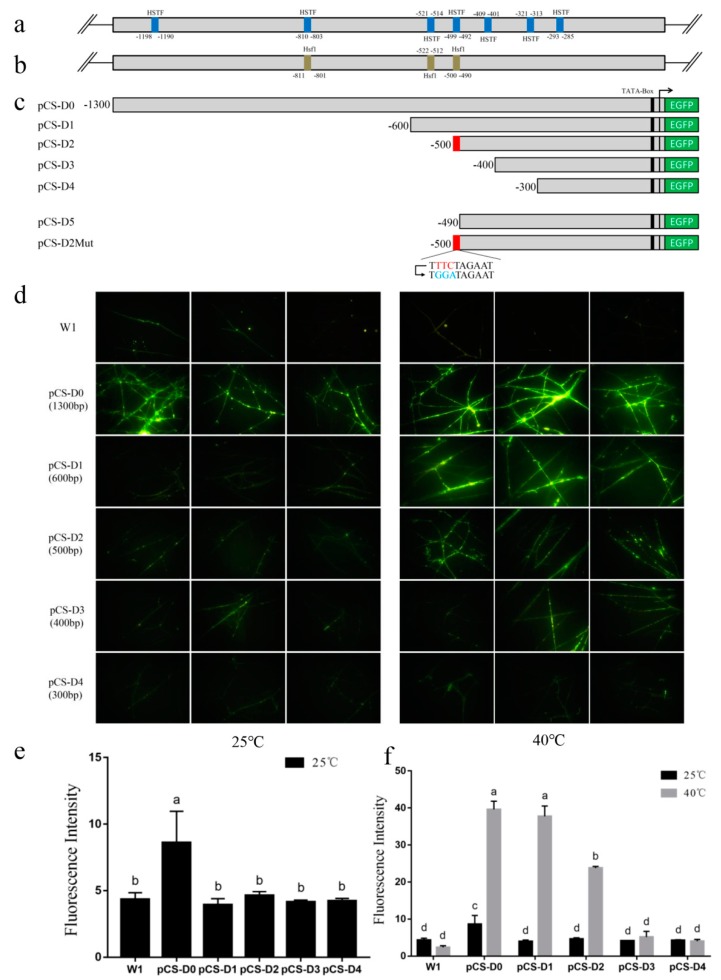
(**a**) HSTF binding sites in *pCS* predicted by SCPD. (**b**) Hsf1 binding sites in *pCS* predicted by Yeastract. (**c**) Construction of expression vectors with the full-length and truncated *pCS*s. (**d**) Mycelia of different transformants before and after heat stress observed with an OLYMPUS BX51 fluorescence microscope compared to wild-type W1; images were taken with a 40× field of view. (**e**) The fluorescence intensity of different transformants under 25 °C; *n* = 3, *p* < 0.01. (**f**) The fluorescence intensity of different transformants before and after heat stress; *n* = 3, *p* < 0.01.

After heat stress, the fluorescence intensity of pCS-D0 transformants significantly increased, whereas the fluorescence intensity of W1 was not changed (Figure 2d,f). Meanwhile, the relative expression level of *Csl* in wild-type W1 showed three-fold higher afterheat stress (Figure 1b). Usually, a constitutive promoter such as the *GPD* (glyceraldehyde-3-phosphate dehydrogenase) promoter can drive gene expression at a high level under any stimuli. However, the promoter *pCS* showed low activity at room temperature, while its activity increased after heat stress. Thus, the promoter *pCS* is suggested to be a heat-inducible promoter in *L. edodes*.

### 2.3. Deletion Analysis of the pCS.

To explore the heat-inducible element that responds to heat stress, a series of 5′deletion constructs were undertaken and transferred into mycelia (W1). As shown in Figure 2, the wild-type W1 strain and pCS-D1,pCSD2, pCS-D3, pCS-D4 transformants showed no green fluorescence, but the fluorescence was obvious in pCS-D0, under normal cultivated condition (25 °C). The full-length fragment of *pCS* (pCS-D0) showed the highest transcriptional activity, while the deletion fragment of *pCS* did not improve the transcriptional activity significantly. Previous studies reported that the highly active fragment of *GPD* gene promoter of 795 bp in *Pleurotus ostreatus* [20], 442 bp in *L. edodes* [21], and 630 bp in *Aspergillus terreus* [22], were more efficient than the full-length of *GPD* gene promoter. Therefore, we concluded that the deletion fragments of *pCS* may not be more efficient than the full-length promoter.

After heat shock for 24 h, the fluorescence intensity of pCS-D0, pCS-D1, and pCS-D2 transformant mycelia significantly increased; however, the pCS-D3 and pCS-D4 transformants showed no significant change. The results indicated that *pCS* was an inducible promoter which can respond to heat stress and the heat-inducible element could be located between −500 bp and −400 bp. The fluorescence intensity in all truncated constructs showed that the predicted HSTF binding sites, which were located between +891 bp and +1015 bp and +102 bp and +497 bp, are not the functional element. Combined with the results of the bioinformatic analysis of the full-length *pCS*, two heat shock transcription factor (HSTF or Hsf1) binding domains were exactly located in this region (−490 bp to −500 bp and +891 bp to +899 bp), but only one of them was predicted by SCPD and Yeastract simultaneously (−490bp to −500bp). Then, it was considered as the supposed heat-inducible element.

### 2.4. Site-Directed Mutation Analysis

**Figure 3:**
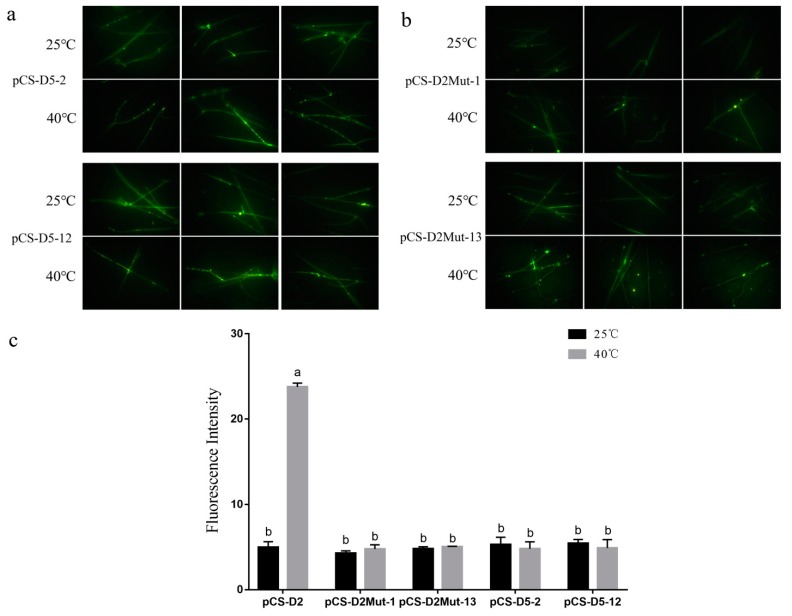
(**a**) Mycelia of pCS-D5 transformants before and after heat stress observed with an OLYMPUS BX51 fluorescence microscope; images were taken with a 40× field of view. (**b**) Mycelia of pCS-D2Mut transformants before and after heat stress observed with an OLYMPUS BX51 fluorescence microscope; images were taken with a 40× field of view. (**c**) The fluorescence intensity of pCS-D5 and pCS-D2Mut transformants before and after heat stress; *n* = 3, *p* < 0.01.

To verify the element within a −490 bp to −500 bp region that was responsible for heat shock, further truncation to −490 bp (pCS-D5) and mutation of this element (pCS-D2Mut) were conducted. As shown in Figure 3, the fluorescence intensity of pCS-D5 transformants mycelia showed no significant change after heat shock, while pCS-D2 transformants did, which meant the deletion of this element lead *pCS* to fail to respond to heat stress. Meanwhile, the pCS-D2Mut transformants also showed no significant change. The difference between pCS-D2Mut and pCS-D2 was only three base-pairs in the supposed heat-inducible element, but completely changed the transcriptional activity. Generally, this 10 bp between -490 bp and -500 bp (TTTCTAGAAT) was the key element required for heat response in *pCS*. Similar result was also reported for the HSE (heat shock element) in the *HSP26* gene of *Saccharomyces cerevisiae* [23]. Moreover, the results also indicated that the one HSTF binding site located between +891 bp and +899 bp was not the functional element, which still appeared in the pCS-D5 and pCS-D2Mut constructs.

## 3. Discussion

**Figure 4:**
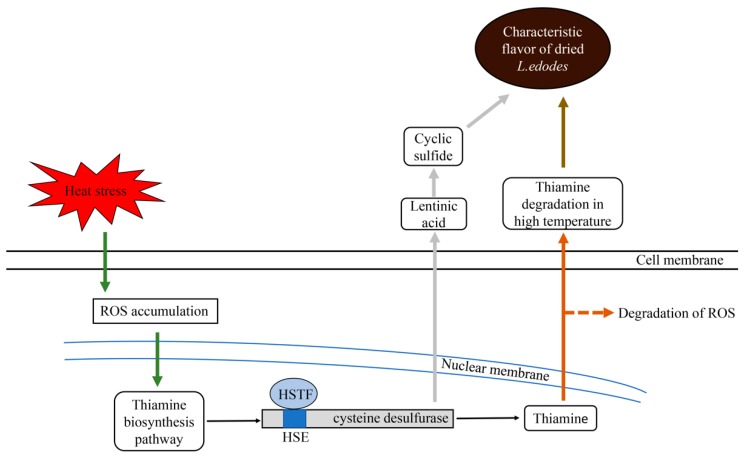
Schematic model of the formation of volatile sulfide during the hot-air drying process. The dotted arrows indicate the result is being explored. **ROS** (reactive oxygen species).

*Lentinula edodes* is a delicious edible mushroom, with a unique volatile flavor, but the mechanisms of the volatile sulfide generation by heat stress is still at the protein level. The main contribution of this work is to advance the mechanisms of flavor formation at the molecular level. Combining the results of this paper, we speculate that the mechanisms of the volatile sulfide formation in the process of hot-air drying of *L. edodes* is as follows: In the early stage of the hot-air drying process, the water activity remains at a high level and the metabolic process still proceeds. It has been reported that the oxidative stress response was stimulated by high temperature during the early stage [24]. We suggest that cysteine desulfurase is activated by the oxidative stress because it is the first enzyme in the thiamine metabolic pathway while thiamine is closely related to the oxidative stress [25,26]. Thiamine metabolic pathway is activated to reduce the damage to mycelia under the oxidative stress. In this process, the HSTF binds with the heat-inducible element which locates between −500 bp and −490 bp in *pCS* and enhances the transcriptional activity of the *Csl* gene, causing the accumulation of cysteine desulfurase. Subsequently, with the temperature rise and the water desorption, a large number of enzymatic reactions occur to generate volatile sulfide, including the reaction catalyzed by cysteine desulfurase with lentinic acid as the substrate. In the later stage of the drying process, non-enzymatic reactions such as a Maillard reaction occurs. Thiamine can degrade and generate volatile sulfide as well [27]. All these reactions contribute to the characteristic flavor for *L. edodes*. The proposed model of the formation of volatile sulfide generated by cysteine desulfurase during the hot-air drying process is shown in Figure 4.

Additionally, the element between−500 bp and −490 bp was the functional heat-inducible element that responded to heat stress, and not the element which was located between, −409 bp and −401 bp, −321 bp and −313 bpand −293 bp and −282 bp. The fluorescence intensity of pCS-D0 transformants after heat stress was 4.6-fold higher than before and 9.6-fold in pCS-D1, and 4.8-fold in pCS-D2. The fluorescence intensity was nearly the same in the pCS-D0 and pCS-D1 transformants after heat stress, but was significantly reduced in pCS-D1 under 25 °C. Therefore, these two elements (−1198 bp to −1190 bp and −810 bp to −803 bp) possibly bound the HSTF during normal conditions to maintain the basal expression level of the *Csl* gene. Monnerjahn and Grosst reported that the HSF was also bound to HSE under normal conditions in *Neurospora crassa*, as well as *Saccharomyces cerevisiae* [28,29]. Furthermore, the predicted HSTF/Hsf1 binding site between −522 bp and −512 bp may be also a heat-inducible element which can bind with one HSTF and improve the transcriptional level of *Csl* after heat stress. It has yet to be further investigated in subsequent research work.

Interestingly, three coincident HSTF/Hsf1 binding domains and one HSTF binding domain (+102 bp to +110 bp) shared the same signal sequence (NTTCNNGAAN), while the other three HSTF binding domains showed totally different signal sequence (GAANNNTCC) [30,31]. Previous studies indicated that HSTFs can bind DNA with the sequence NGAANNTTCN or with the sequence NTTCNNGAAN in yeast [32]. These two sequences are also called heat shock elements. Our research showed that the sequence of the elements in *pCS* can respond to heat stress the same as the heat shock elements.

In *pCS*, there were three or six conceivable HSEs, but only one could respond to heat stress, which meant not all the HSEs can bind with HSTFs/Hsf1 or only several HSTFs/Hsf1 can bind with the promoter region. Young and Craig [33] found that the *Hsp70* gene *SSA1* had multiple heat shock elements, but only two of them were active promoter elements [33]. Chen and Pederson [23] also found that the rate of response to heat stress was correlated with the HSF occupancy of HSEs, rather than the number of HSEs in the promoter [23].

In sum, the heat-inducible element in the promoter *pCS* can be a new target site for the flavor quality improvement and provide a deep understanding of the formation of flavor in *L. edodes*.

## 4. Materials and Methods

### 4.1. Strains and Culture Conditions

The wild-type strain *L. edodes* W1 (collected in Institute of Applied Mycology, College of Plant Science and Technology) used as the recipient host strain for transformation was grown on MYG medium (1% malt extract, 0.1% peptone, 0.1% yeast extract, 2% glucose) at 25 °C. The DH5α strain of *Escherichia coli* used for plasmid amplification was grown on Luria–Bertani (LB) medium containing 100 μg/mL kanamycin at 37 °C. The EHA105 strain of *Agrobacterium tumefaciens* used for fungal transformation was grown on Luria–Bertani (LB) medium containing 100 μg/mL kanamycin and 50 μg/mL rifampicin.

### 4.2. Extraction of Genomic DNA

The mycelium was grown on cellophane membranes laid over MYG medium containing 5 μg/mL Hyg for 10 days at 25 °C. Then, the mycelium was collected and ground to powder in liquid nitrogen. For DNA extraction, the mycelium powder was mixed with extraction solution including 475 uL CTAB (Hexadecyl trimethyl ammonium Bromide, 100 mM Tris-HCl (pH = 7.8), 20 mM EDTA (Ethylenediaminetetraacetic acid), 1.4 M NaAc, 2% (*w*/*v*) CTAB), and 25 μL 10% SDS (Sodium dodecyl sulfate) [34]. Then the tube was vortexed for 30 s and mixed with 500 μL PCI (Phenol:chloroform:isoamyl alcohol = 25:24:1, *v*/*v*). After being centrifuged at 12,000 rpm for 10 min, the supernatant was collected into a new centrifuge tube and mixed with 3/4 volume isopropyl alcohol. Then, after being centrifuged at 12,000 rpm for 5 min, the precipitate was washed twice with 70% ethanol and dissolved with 100 μL distilled water.

### 4.3. Cloning and Analysis of Full-Length pCS

According to the genome information of *L. edodes* v1.0 [35], we cloned the 1300 bp up-stream of the initiation codon ATG in the *Csl* gene as the promoter region. The promoter sequence was amplified from the extracted genomic DNA of *L. edodes* W1 strain in a 50 μL reaction system via PCR (3 min at 95 °C, 35 cycles of 15 s at 95 °C, 15 s at 60 °C, 70 s at 72 °C, and 5 min at 72 °C) with paired primer pCS-R/pCSD0-F. This promoter region was analyzed online by SCPD (The Promoter Database of *Saccharomyces cerevisiae*, http://rulai.cshl.edu/SCPD/) and Yeastract (Yeast Search for Transcriptional Regulators and Consensus Tracking, http://www.yeastract.com/index.php) to predict the possible cis-acting elements as well as transcriptional factor binding sites.

### 4.4. Vector Construction of the pCS and Its Deletion Derivatives

The full-length promoter and its 5′deletion fragment were amplified from genomic DNA with primers in Table 3 via PCR reaction. The gene *EGFP* was amplified from the vector pCAMBIA1300-EGFP (constructed at the Institute of Applied Mycology, College of Plant Science and Technology) via PCR reaction. All PCR products were analyzed by agarose gel electrophoresis and collected with SanPrep Column DNA Gel Extraction Kit (Sangon Biotech, Shanghai, China). The pCAMBIA1300-g vector was digested with EcoR I and Kpn I in a 50 μL reaction system. Then, the promoter fragment, *EGFP*, and the linearized pCAMBIA1300-g vector were linked together by homologous recombination to generate the promoter expression construct. For the mutation construct, the mutant fragment was simultaneously digested by EcoR I and Kpn I and then linked with linearized pCAMBIA1300-g backbone by T4 DNA ligase. Six promoter expression plasmids were then transformed into *Agrobacterium tumefaciens* strain EHA105.

### 4.5. Site-Directed Mutation and Further Truncation

Based on the results of the truncated transformant mycelia fluorescence intensity as well as *cis*-acting element prediction of *pCS*, 10 bp (TTTCTAGAAT) located within −490 bp to −500bp were suggested to be the conceivable element responding to heat stress. Then, the homologous recombination primers were designed to introduce 3 bp sites mutation (TTTCTAGAAT changed into TGGATAGAAT) to this 10 bp. The forward primer contained a restriction enzyme cutting site of EcoR I and the reverse primer contained a site of Kpn I.

### 4.6. Agrobacterium-Mediated Fungal Transformation

Agrobacterium-mediated transformation of *Lentinula edodes* was performed as described by Wang Gang Zheng [34]. The *A. tumefaciens* strain EHA105 containing the promoter expression plasmid was cultivated at 28 °C with shaking at 200 rpm in MM (Minimal Medium) for 2 days. The *Agrobacterium tumefaciens s* cells were collected and suspended with IM (Induced Medium) to an OD600 around 0.4 and incubated for 6 h at 28 °C with shaking at 200 rpm. Mycelia of *L. edoses* were grown on a MYG plate for 2 days and then infected by pre-induced *A. tumefaciens* for 20 min and placed on Co-IM (co-cultivated induced medium) for 2 days at 28 °C to complete the fungal transformation. After 2 days of co-cultivation, the mycelia were transferred to selection medium containing 3 μg/mL hygromycin B and 200 μg/mL cefotaxime to screen the possible transformants, then continuously transferred the mycelia twice on selection medium containing hygromycin B. The transformants were detected by PCR reaction using primers EGFP-F/R and hph-F/R to identify the band of the *EGFP* and *hph* genes, respectively.

### 4.7. Fluorescence Microscopy and Quantification of EGFP Fluorescence Intensity

Two positive *L. edodes* transformants were randomly selected and cultivated on MYG medium containing 5 μg/mL hygromycin B for 5 days until the mycelia were grown on the glass slides, while the wild-type W1 was cultivated without hygromycin B. The mycelia were observed under an OLYMPUS BX51 fluorescence microscope (OLYMPUS, Tokyo, Japan) with excitation at 455–490 nm to examine *EGFP* expression before and after heat shock (40 °C, 24 h). Images were taken with a 40× field of view. The imaged area was selected randomly, but clearly because the mycelia were grown irregularly. Each transformant was imaged in three different perspectives and the fluorescence intensity was the average value of these three perspectives. The numerical value of fluorescence intensity was calculated by Image-Pro Express (Media Cybernetics, Rockville, MD, USA). The *p*-value was calculated with the Duncan test and the error-bars in figures representing SD. The letters above the bars in figures represent different groups with highly significant differences (*p* < 0.01).

## Figures and Tables

**Table 1:** The transcription factor binding sites predicted by SCPD.

Element Name	Signal Sequence	Putative Function	Numbers
GCN4	TGAATA	Transcriptional activator binding site	17
HSTF	TTCAACGAA	Involved in heat response	7
REB1	CCACCCG	RNA polymerase IIbinding site	1
ECB	GGAAAAA	Early cell-cycle box element	1
ADR1	TCTCC	Transcriptional activator binding site	3
GCR1	CTTCC	Involved in decomposition of sugar	7
CuRE	GAGCAAA	Cu^2+^ response element	1
PHO4	CACGTT	Activation of phosphate metabolism related genes	2
ABF1	TCATTCCAGACG	Transcriptional activation of numerous genes	1
TBP	TATATA	TATA-Box binding protein	2
UASPHR	CTTCCT	Involved in DNA repair	1
STRE	AGGGG	Involved in stress response	1

**Table 2:** The transcription factor binding sites predicted by Yeastract.

Element Name	Signal Sequence	Putative Function	Numbers
Ash1	YTGAT	Transcriptional inhibition of *HO* gene	9
Cat8, Sip4	NCCDTYNVNCCNG	Involved in the rearrangement of carbon metabolism	1
Crz1	GNGGCKCA	Involved in calcineurin activation	1
Fkh1, Fkh2	RYMAAYA	Involved in cell cycle and differentiation	3
Gat1, Gln3, Gzf3	GATAAG	Involved in glyceride metabolism	1
Gcn4	TGATTCA	Activating amino acid synthesis related genes	1
Gcr1	CTTCC/CWTCC	Regulation of glycolytic related genes	9
Gis1, Msn2, Msn4, Rph1, YER130C	AGGGG	Regulation of diphosphate pyrophosphate metabolism	1
Hsf1	NTTCNNGAAN	HSTF binding site	6
Mac1	TTTGCKCR	Cu^2+^ response element	1
Mot3	AAGGWT	Involved in oxygen stress	8
Msn2, Msn4, Rph1	CCCTC	Involved in stress response	1
Nrg1	CCCTC	Regulates glucose metabolism and response to alkali	2
Pho4	CACGTK	Response to phosphate limitation	2
Rgt1	CGGANNA	Regulation of multiple glucose transporter genes	1
Rtg1, Rtg3	GTCAC/GGTAC	Involved in interorganelle communication	2
Skn7	GGCCAGA	Response to oxidative stress and osmoregulation	1
Stb5	CGGNS	Regulating multidrug resistance and oxidative stress response	15
Tec1	CATTCT	Regulating hyphal growth	2
Xbp1	CTCGA	Cyclin gene transcriptional repression	2
Yap1	TGACAA	Required for oxidative stress	2
Rim101	TGCCAAG	Response to pH and in cell wall construction	2
Haa1	SMGGSG	Involved in adaptation to weak acid stress	3
Com2	ATAGGGT	Involved in adaptation to stress	1

**Table 3:** Sequence of the primers.

Primer Name	Primer Sequence(5′-3′)
pCS-R	ccttgctcaccatGTTCAGTTAATCAAGGGGGTGAGG
pCSD0-F	tctagaggatccccgggtaccATGGGTGAATATAGAGAGGCGG
pCSD1-F	tctagaggatccccgggtaccCTGTAGCAGATTCTGAAAAGATTGTAGC
pCSD2-F	tctagaggatccccgggtaccTTTCTAGAATCAGTTTGATTCAGGTCTG
pCSD3-F	tctagaggatccccgggtaccTGAGATCTCATGCTACAGTGTGCA
pCSD4-F	tctagaggatccccgggtaccAGGTAAGGAACTGTCCTTGATTTCA
pCSD5-F	tctagaggatccccgggtaccCAGTTTGATTCAGGTCTGATTCGG
EGFP-F	actgaacATGGTGAGCAAGGGCGAGG
EGFP-R	ccacctcaaacttcggaattcTTACTTGTACAGCTCGTCCATGCC
hph-F	TCGTCCATCACAGTTTGCC
hph-R	TGCCTCTAATCCCTTGCTC
qEGFP-F	AAGGGCATCGACTTCAAGGAG
qEGFP-R	GTTCACCTTGATGCCGTTCTTC
pCSD2Mut-F	GCCGAATTCTGGATAGAATCAGTTTGATT
pCSD2Mut-R	GGTACCTTACTTGTACAGCTCGTCCAT
